# The role of urgent care centers in headache management: a quality improvement project

**DOI:** 10.1186/s12913-021-07457-2

**Published:** 2022-02-08

**Authors:** Mia T. Minen, Dennique Khanns, Jenny Guiracocha, Annika Ehrlich, Fawad A. Khan, Ashhar S. Ali, Marius Birlea, Niranjan N. Singh, Addie Peretz, I. V. Larry Charleston

**Affiliations:** 1 Departments of Neurology and Population Health, NYU Langone Health, 222 East 41st Street, 9th floor, New York, NY 10017 USA; 2 City College, CUNY, New York, NY USA; 3 UCSF Headache Center, University of California, San Francisco, CA USA; 4 UCSF School of Nursing, San Francisco, CA USA; 5 The McCasland Family Comprehensive Headache Center, Ochsner Neuroscience Institute, Ochsner Clinic Foundation, New Orleans, LA USA; 6 The University of Queensland School of Medicine, Ochsner Clinical School, New Orleans, LA USA; 7 Tulane University School of Medicine, New Orleans, LA USA; 8 Henry Ford Health System, Department of Neurology, Division of Headache, Detroit, MI USA; 9 Wayne State University School of Medicine, Detroit, MI USA; 10 Department of Neurology, University of Colorado Anschutz Medical Campus, Aurora, CO USA; 11 Neurology, University of Missouri, Columbia, MO USA; 12 St Mary’s Stroke program, St. Mary’s Regional Medical Center, Blue Springs, MO USA; 13 SSM Neurosciences Institute, SSM Health, St. Louis, MO USA; 14 Department of Neurology, Division of Headache and Facial Pain, Stanford University, Palo Alto, CA USA; 15 Michigan State University College of Human Medicine, East Lansing, MI USA

**Keywords:** Urgent care visits, Headache, Migraine, Infusion therapy, Administration

## Abstract

**Background:**

Patients with headache often seek urgent medical care to treat pain and associated symptoms that do not respond to therapeutic options at home. Urgent Cares (UCs) may be suitable for the evaluation and treatment of such patients but there is little data on how headache is evaluated in UC settings and what types of treatments are available. We conducted a study to evaluate the types of care available for patients with headache presenting to UCs.

**Design:**

Cross-Sectional.

**Methods:**

Headache specialists across the United States contacted UCs to collect data on a questionnaire. Questions asked about UC staffing (e.g. number and backgrounds of staff, hours of operation), average length of UC visits for headache, treatments and tests available for patients presenting with headache, and disposition including to the ED.

**Results:**

Data from 10 UC programs comprised of 61 individual UC sites revealed: The vast majority (8/10; 80%) had diagnostic testing onsite for headache evaluation. A small majority (6/10; 60%) had the American Headache Society recommended intravenous medications for acute migraine available. Half (5/10) had a headache protocol in place. The majority (6/10; 60%) had no follow up policy after UC discharge.

**Conclusions:**

UCs have the potential to provide expedited care for patients presenting for evaluation and treatment of headache. However, considerable variability exists amongst UCs in their abilities to manage headaches. This study reveals many opportunities for future research including the development of protocols and professional partnerships to help guide the evaluation, triage, and treatment of patients with headache in UC settings.

**Supplementary Information:**

The online version contains supplementary material available at 10.1186/s12913-021-07457-2.

## Background

Migraine, a chronic disabling condition characterized by acute attacks of head pain and associated symptoms, accounts for a substantial portion of the 4–5 million emergency department (ED) visits per year for headache [1, 2]. Long wait times, loud noises and bright lights, overuse of neuro-imaging, and suboptimal treatment of acute migraine attacks with medications such as opioids, make the ED less than ideal for patients with migraine [3]. Headache specialists, in turn, have employed infusion centers as a mechanism to prevent ED visits. However, a recent study of headache infusion centers showed that few centers offer infusions outside business hours [4]. Moreover, headache providers cited numerous barriers to maintaining these centers [4].

An alternative solution for treating patients with refractory migraine may be the use of urgent care (UC) facilities, sometimes known as Walk-in Clinics or Express Care clinics. Urgent Care Services are defined by the Centers for Medicare and Medicaid Services (CMS) as services furnished within 12 h in order to avoid the likely onset of an emergency medical condition. UC facilities location is distinct from a hospital emergency room, an office, or a clinic, and purpose is to diagnose and treat illness or injury for unscheduled, ambulatory patients seeking immediate medical attention [5, 6]. Currently, there are over 8000 UC facilities in the United States, with a 58% UC growth rate from 2013 to 2019 [7, 8]. UC facilities are designed to manage unplanned visits for lower acuity conditions [9]. They are widely available and offer same-day and walk-in appointments after hours and on weekends, an ideal circumstance for the needs of patients with migraine. UC facilities result in cost savings by decreasing unnecessary ED visits and a concomitant increase in hospitalizations [10]. Statistics demonstrate that 14–27% of ED visits could be handled in an alternative medical setting like an UC facility [9]. One financial evaluation showed that a switch of these cases to UC facilities could result in savings of up to $4 billion per year [10].

While two recent studies examined UCs and migraine in New York City [11, 12], they only looked at the number of UC visits for headache and/or migraine in an 8-month period (over 10,000) [12] and migraine management in UC facilities that were part of one urban academic medical center. In the latter study, we learned that there are ways UC facilities might be optimized to treat people with migraine, i.e. stocking migraine specific medications like sumatriptan, having pain assessments for those complaining of pain so that providers can assess whether pain decreases on discharge, considering the use of headache protocols and tools like the Migraine Action Plan [13] which can guide providers as to which medications to use, and ensuring that medications for various migraine symptoms (i.e. nausea/vomiting) are prescribed when needed upon discharge. In this study, we seek to assess UC facilities’ headache management practices at multiple sites across the US, how they compare to previously studied UC facilities, and to identify potential opportunities to improve headache care in the UC setting.

## Methods

We submitted to the Institutional Review Board (IRB) of New York University Langone Health where it was deemed a quality improvement study and thus IRB approval was not needed. In an effort to compare other UC facilities to the one published study on UC for migraine [11] and to better understand how UC facilities might serve the headache population, an email invitation was sent to headache specialists who are members of the American Headache Society Refractory/Inpatient/Emergency Care Special Interest Section to (1) ask them what questions they would like to ask on a survey and (2) whether they would like to help collect data for the study. The survey was then written using an iterative approach over email based on the findings of the prior study [11] to understand the operations specific to their local UC facilities with specific questions targeted to headache practice and treatment. Facilities that fulfilled the CMS definition for urgent care services or facilities were selected by convenience sampling. Local UC centers were either known to authors, located via web searches or health provider recommendations. The full questionnaire can be found in the Additional file 1: Appendix.

These authors then contacted their local UC facilities by phone and/or email and collected the data. The eight headache specialists on the author panel obtained the information in the following ways: 3 headache specialists (AE, AP, FK) emailed the survey questions and headache specialists (AA, AE, LC, MB, MTM, NS) asked the questions by phone. In a few cases, (MTM) they received emails confirming some of the responses. The headache specialists communicated with a range of professionals who could provide the necessary information. Three headache specialists (AA, FA, MTM) contacted the medical directors of the urgent care centers, one spoke with a Clinical Associate Professor of Primary Care (AP), one (AE) spoke with the Clinical Nurse Manager and sent an email with follow-up questions to the medical director, another spoke with a NP (LC) and then two others (MB, NS) reported speaking with a range of people, depending on the availability and knowledge: providers (most often advanced practice provider), medical assistant, receptionist (most commonly), practice manager, other staff.

Of note, for the purposes of this study, a UC facility could be defined as an “urgent care,” “express care,” or “walk in” facility.

The data obtained from the local UC facilities was recorded in Redcap [14] and descriptive statistics were conducted in Excel. We report means, medians, percentages, and standard deviations.

## Results

**Fig. 1:**
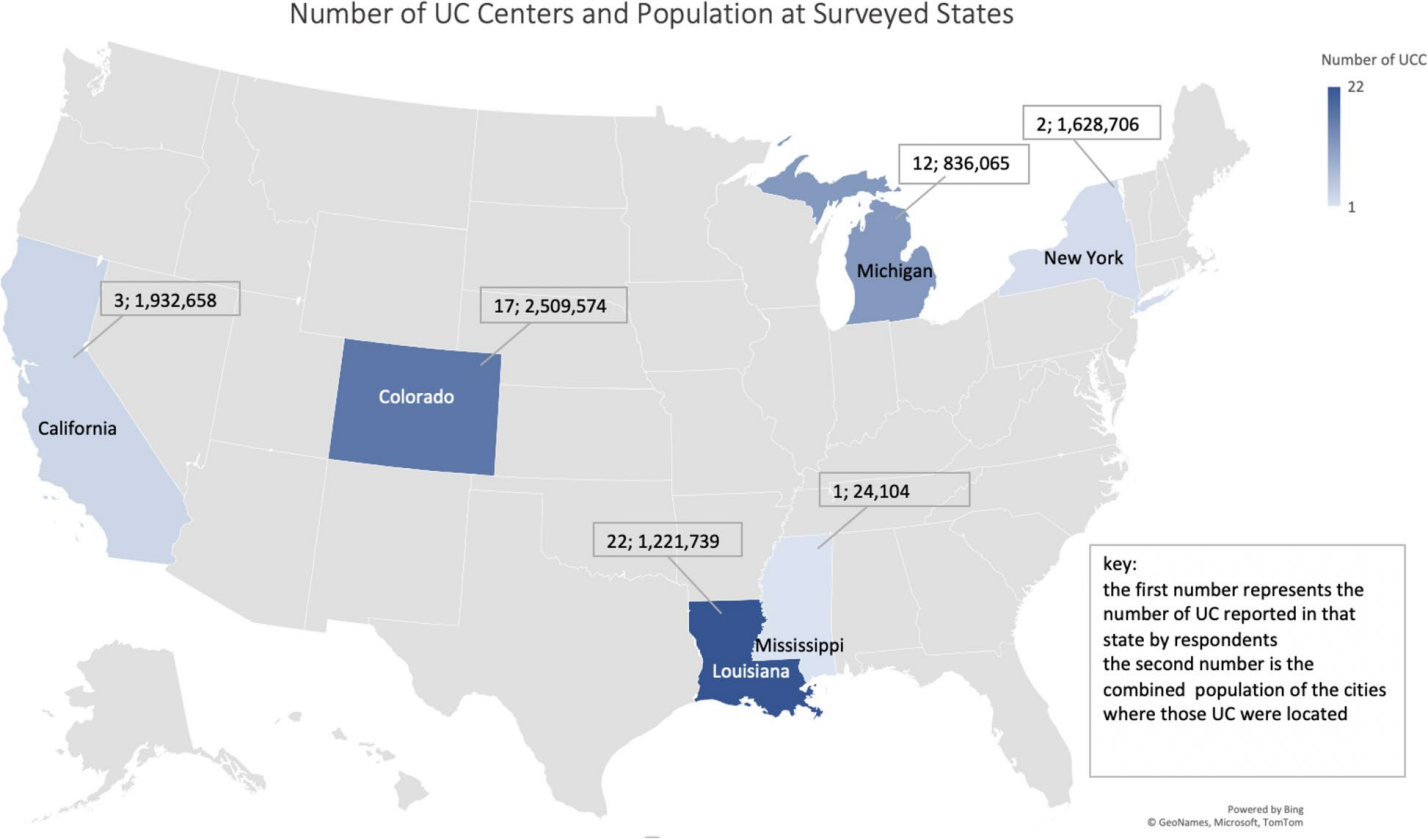
Number of UC Centers and Population at Surveyed States. Created using Microsoft Excel software, freely available to use

Between June 2020 and July 2020, a total of eight headache specialists contacted their local UC programs and collected and entered the data for 10 different UC programs in the US. The 10 UC programs comprised 61 individual UC facilities. Within each UC program, there was a mean of 6 UC sites and a median of 2 UC sites. The UC programs were scattered around the country (See Fig. 1: Map). Of the 61 individual UC sites reported, the majority (56%, 34/61) were part of an institution and most of the remaining (41%, 25/61) were free standing sites. Two sites (3%, 2/61) were identified as both freestanding and part of an institution. As noted in Table 1, an average of 41,621.6 total visits per year for all conditions were reported at the surveyed UC programs. The average reported number of physicians, nurse practitioners, and physician assistants per UC program was 12.8, 3.1, and 1.8 respectively.

**Table 1:** Characteristics of Surveyed Urgent Care Facilities

Question		N
*Number of different urgent care locations*		*N* = 61
	(mean^a^,sd)	(6.1,7.4)
	range	{1, 2}
*Specific area of the urgent care location provided*^*b*^		*N* = 10
	Urban	50% (5)
	Suburban	40% (4)
	Rural	10% (1)
*Number of visits/year*		*N* = 416,216
	(mean,sd)	(41,621.6,92,464.8)
	range	{50, 300,000}
*Affiliation*		
Free Standing	Free standing	41% (25)
Part of Medical Institution	Part of medical institution	56% (34)
Both	Both	3% (2)
*Staffing*		
Number of Physicians per urgent care site		*N* = 12.8
	(mean,sd)	(12.8,14.2)
	range	{0, 40}
*Number of Nurse Practitioners/urgent care site*^*c*^		*N* = 3.1
	(mean,sd)	(3.1, 2.5)
	range	{0, 7}
*Number of Physician Assistants/urgent care site*		*N* = 1.8
	(mean,sd)	(1.8, 2.0)
	range	{0, 6}
*Number of Nurses/urgent care site*		N = 3.1
	(mean,sd)	(3.1, 5.5)
	range	{0, 17}
*Number of Medical Assistants/urgent care site*		*N* = 5.7
	(mean,sd)	(5.7, 8.8)
	range	{0, 30}

^a^This is the average number of locations provided per respondent

^b^Respondents provided more than one UC location in the area where their US is located but only provided the specific area for the UC they provided an address for

^c^5 out of 10 respondents answered that there were no nurse practitioners at the site listed

For the remainder of the Results section (including the tables), all reported data are based on the 10 UC programs in aggregate.

UC programs reported that the most common medical specialty training for their physicians (either MD or DO), was family medicine (80%, 8/10), emergency medicine (70%, 7/10), and internal medicine (60%, 6/10). There were prior work requisites reported for most programs: 8 UC programs (80%, 8/10) reported a minimum of 1 year of experience in internal medicine or family medicine (10%, 1/10), at least 2 years of emergency experience (10%, 1/10), 1–2 years of outpatient experience (10%, 1/10), Accreditation Council for Graduate Medical Education (ACGME) residency and board certification in primary specialty (30%, 3/10), completion of residency (10%, 1/10), and at least 2 years of urgent care management after residency completion (10%, 1/10). Table 3 further shows the number of UC programs who listed prerequisites for nurse practitioners (50%, 5/10) and physician assistants (40%, 4/10).

Hours of operation varied with (20%, 2/10) remaining open for greater than 12 h on Monday through Friday and 70% (7/10) open on two weekend days per week (Table 2). Each UC program had the capacity to offer, on average, 10 medications; ondansetron (100%, 10/10), acetaminophen PO (70%, 7/10), and ibuprofen PO (70%, 7/10) were the most common medications reported as treatment options for migraine. Regarding the highest-level recommended drugs (Level B) per the American Headache Society’s (AHS) emergency department migraine management guidelines [12], 50% (5/10) reported using subcutaneous sumatriptan, 60% (6/10) reported using intravenous (IV) metoclopramide, and 50% (5/10) reported using intravenous prochlorperazine. Across the 10 UC programs, 9 (90%, 9/10) have staff available to administer IM medications and 6 (60%, 6/10) to administer IV medications. Those same 6 UC programs (60%, 6/10) can administer both IM and IV medications. Specifically, 6 programs offer IV metoclopramide (60%, 6/10), while 5 programs offer IV prochlorperazine (50%, 5/10). Of those 5, 4 programs (40%, 4/10) offer both IV metoclopramide and prochlorperazine. UC programs surveyed attested to the use or pharmacy storage of Morphine IV (4/10), Morphine PO (1/10), and Hydromorphone IV (2/10), Acetaminophen/Codeine (2/10). All other medications available are listed on Table 4.

**Table 2:** Hours of Operation at the Urgent Care Locations Provided

Hours of Operation	N
*Open Monday to Friday*	N = 10
< 12 h {7:30 am -8 pm}	40% (4)
12 h {8 am -9 pm}	40% (4)
> 12 h (4 am-8 pm, 24 h)	20% (2)
*Weekend hours*	*N* = 10
Open one weekend day	30% (3)
Open two weekend days	70% (7)
*Reported average length of stay for migraine and headache patients*^a^	N = 10
< 1 h	30% (3)
1–2 h	40% (4)
> 2	20% (2)

^a^one respondent left question blank

**Table 3:** Examples of Prerequisites Needed To Work at Urgent Care Locations Provided

Question	Providers’ Responses^a^
	N = 4
*What are the prerequisites for Physician Assistants to work at the site?*	Trained with family medicine or internal medicine Graduate from a Physician Assistant program 1 year of urgent care experience after PA certification 2 years of urgent care experience after PA certification
	N = 5
*What are the prerequisites for Nurse Practitioners to work at the site?*	At least 2 years of nursing background in critical care or EM (*n* = 2) Must have FM or IM training Graduation from Nurse practitioner program Nurse Practitioner Master’s Degree, board certified – 1 year on the job fellowship training
	*N* = 8
*What are the prerequisites for Physicians to work at the site?*	At least 1 year of experience and must be IM or FM doctor Must have 1–2 years of outpatient experience At least 2 years of emergency experience Must have ACGME residency and board certification in primary specialty (*n* = 3) Complete residency and have at least 2 years of urgent care management after residency completion

^a^The “n” varied because not all sites had all types of providers i.e. some sites did not have nurse practitioners or physician assistants. Thus, this question was not applicable for some sites

**Table 4:** Medication Administration at Urgent Care Locations Provided

Question	N
*Are there staff available to administer the following medications?*	N = 10
IM	90% (9)
IV	60% (6)
Both	60% (6)
*Which migraine medication(s)/antiemetic(s) are kept in the pharmacy? (multiple selections allowed)*	N = 10
Metoclopramide IV	60% (6)
Prochlorperazine IV	50% (5)
Both	40% (4)
Metoclopramide PO	60% (6)
Depakote IV	20% (2)
Dihydroergotamine (DHE) IV	10% (1)
IVF	50% (5)
Diphenhydramine IV	40% (4)
Magnesium IV	30% (3)
Ketorolac IV	50% (5)
Dexamethasone IV	50% (5)
Ondansetron (PO)	100% (10)
Sumatriptan inj	50% (5)
Oral triptans	50% (5)
Morphine IV	40% (4)
Morphine PO	10% (1)
Hydromorphone IV	20% (2)
(Oxycodone/Acetaminophen)/Acetaminophen/Codeine	20% (2)
Ibuprofen PO	70% (7)
Naprosyn PO	40% (4)
Acetaminophen IV	10% (1)
Acetaminophen PO	70% (7)
Ketamine IV	0% (0)
Ketamine NS	0% (0)
Other^b^	6% (6)
*Average number of medications per UC program*	10

^b^Participants specified medication used under protocol: Sumatriptan (injectable), avoiding opioids, dihydroergotamine, isometheptene, Benztropine, Lorazepam, Antiemetics, IV NSAIDs, IV Ergots, Antiepileptics, Haloperidol, Ketorolac, Opiates, Methylprednisolone, Dexamethasone, IVF, Ondansetron, Diphenhydramine, Topiramate, Calcitonin gene related peptide monoclonal Antibodies, onabotulinum toxin, triptans, gepants, ditans, neuroleptics

^b^includes Toradol IM, oral steroids, NSAID PO,IM, ketorolac IM, Ketoprophen, Metamizol, Tramadol, Chlorpromazine, IV caffeine for spontaneous intracranial hypotension

**Table 5:** Protocol for Treating Headaches and Migraines at Urgent Care Locations Provided

Question	N
*Is it regular practice for providers (any) at your urgent care to do pain checks?*	N = 10
Yes	80% (8)
*Pain assessments used in evaluating and managing patients presenting with headache to the urgent care(s)*	N = 8
VAS	50% (4)
Wong-Baker FACES Pain Rating Scale	12.5% (1)
Pain assessment screener	12.5% (1)
Pain scale/numeric rating scale	25% (2)
*Diagnostic test(s) performed onsite for patients with headache disorders (multiple selections)*	N = 8
X-Ray	25% (2)
MRI	25% (2)
CT Scan	37.5% (3)
Labs (bloodwork)	52.5% (5)
Physical Exam only	12.5% (1)
Other (EKG, Sleep Study, Neuroimage)	37.5% (3)
*Clinical diagnostic tool used*	N = 10
ICHD3	10% (1)
EPIC screening tools	10% (1)
*Are there any policies in place to ensure follow-up with a patient’s PCP, neurologist, or headache specialist?*	N = 10
Yes^a^	40% (4)
*What percentage of patients that present with headache have a disposition to the emergency department?*^*b*^	N = 10
*> 1%*	20% (2)
*What type of providers do you refer to? (multiple selections allowed)*	N = 10
Primary Care Physician (PCP)	70% (7)
Neurologist	70% (7)
Headache Specialist	60% (6)
Pain Specialist	40% (4)
Other Healthcare provider^c^	30% (3)
Are there home UC locations in your area?	N = 10
Yes	20% (2)
*Does the home UC treat migraine/headache?*	N = 10
Yes^d^	20% (2)

^a^Referrals sent to Headache Clinic if it is a chronic issue (or 2x visits in 1 year), PCP is always cc-ed on chart and patient is instructed to follow up with PCP or return to urgent care in 2–4 business days or go to ED (discourage ED use); Most often in our practices, patients are referred from their provider to these units. Follow-ups are scheduled or ensured on discharge; Started to schedule patients consultation with a neurologist or headache specialist in the moment of the patient delivery from ED; See all patients back in 4 weeks until significant improvements in headache burden are made. Patients come in more frequently if needed for urgent care

^b^Eight respondents left question blank

^c^Includes ophthalmologists, sleep centers, physical therapists, hormone specialists, endocrine, weight management, ENT, cardiologist

^d^Newly established local Urgent Care (about 1 year) uses typical medication for headache care; excludes IV treatments and opioids; offers telemedicine and consultations with MD/DO; remedy room established to treat patients with migraine/tension headache/hangover headaches

*ICHD3* International Classification of Headache Disorders-3

**Table 6:** Future Directions for Headache Care in Urgent Care

**Educating UC Providers**	-In terms of targeting the specific providers who are most likely to come work in an UC facility, research has shown that most facilities (95.8%) have physicians on staff, and family medicine is the most common specialty (present at about three quarters of the centers) [17]. -Other specialties sometimes staffing them include emergency medicine, internal medicine, and pediatrics. About half also have advanced practice providers (NPs and PAs). -Thus, there is a continuing need for headache education among primary care and emergency physicians, physician assistants, and nurse practitioners. Given its population prevalence and associated disability, headache is inadequately covered in both emergency medicine and primary care residency curricula. Post-residency, management of headache should be a frequent topic of grand rounds and conference-based educational programs. -Initiatives similar to the American Headache Society First Contact-Primary Care Initiative which educated PCPs about migraine [18] might be expanded to include urgent care providers. -The American Academy of Pain Medicine, through its Headache Special Interest Section and its primary care migraine guidelines initiatives, might also help with this effort.
Partnerships with Academic Medical Centers/Neurology Departments/Headache Centers	-There has been a move toward UC facilities partnering with academic healthcare systems as a way of bringing in more patients to the healthcare systems. This has occurred throughout New York City [19, 20]. - These numerous partnerships between UC facilities and big academic health systems can lend themselves to not only UC facilities referring patients appropriate for specialist care, but to partnerships in which neurologists and headache specialists might use these UC facilities to provide acute care e.g. infusion treatments for their headache patients rather than setting up headache specific infusion centers that might require significant staffing needs and/or sending their patients to the ED for such care. This might reduce headache ED repeat visits which have been found to be predominantly due to headache-related acute care [21]. In addition, whereas a prior study found that a substantial number of headache specialists are dissatisfied with the care their patients receive in the ED, in part because they felt that there was little communication between the ED physicians and the primary headache providers [22], such partnerships between UC facilities and neurologists/headache specialists might improve communication between providers in these different settings.
Educating Patients about the Option to Seek Acute Migraine Treatment in UC Facilities	-Future work might educate patients about the difference between care provided at the UC verses the ED, providing a list of nearby UCs, their working hours, resources available and when to triage ED over UC should be a standard part of office visit counseling and coordination of care and should help to off load ED burden by diverting unnecessary patient volume as the patient is more likely to listen to their established provider more than anybody else. -Headache providers might provide patients with an after-hours/weekend protocol e.g. the Migraine Action Plan [23]. -In addition to outpatient medical providers advising patients of these options, if protocols are put into place, school nurses might be able to evaluate and refer students and their families to UC facilities [24].
Examining Patient Decision-Making to Seek Care in ED versus Urgent Care Facilities	-Future work should examine patient decision making in deciding to visit an ED versus an UC facility for headache with a special focus on examination of race, ethnicity, and socio-economic factors. A cross sectional study of Medicare and Medicaid beneficiaries examined predictors of who were more likely to go to UC versus ED for a non-emergent health condition [13], All those examined lived within a 10-mile radius of 12 UCC locations and have had more than one visit to a UCC, emergency department, or both. The authors of that study found low utilization of the UCCs. -Demographically, Black participants were more likely to go to the ED compared to White participants, regardless of how close the UCC was to them and the type of insurance they used [13]. The authors concluded that the Black participants felt more comfortable walking into the ED. Also, although there has been an increase in UC facilities across the US, this growth tends to distribute in locations with higher income and more insured patients [25–28]. -Patients who visit UCs may have better insurance [17] and thus better access to outpatient headache care as well. -A study found that UC facilities may worsen the disparities within healthcare due to financial interest, especially since refusal of service is allowed if funds are not met by the patient [25]. That said, UC facilities tend to be located in areas with a high proportion of individuals from historically marginalized/non-White populations, possibly to help mitigate the disparities associated with race and ethnicity, prompting some to conclude that the decision to locate UC centers is independent of race and fully considerate of economic advantages [25].

Five out of 10 UC programs (50%) mentioned that there was a headache or migraine protocol in place at the respective UC sites. The majority (60%, 6/10) reported that there was no policy in place to ensure that the patient would follow up with their primary care provider (PCP), neurologist, or headache specialist. One of the programs that had a headache treatment protocol in place did not have a follow up policy. Only two programs reported that > 1% patients have a disposition to the ED. The remainder said “none” when asked about patients with headache being referred to the ED from the UC.

Table 5 reports the assessments/protocol used for pain and headache management. A total of 8 UC programs (80%, 8/10) complete pain checks regularly and reported having pain assessments at their corresponding UC program to evaluate and manage patients with headaches. Onsite diagnostic test(s) for patients with headache disorders are provided at 8 (80%, 8/10) of UC programs. Out of the 9 programs that provided the average length of stay for patients with headache, 3 programs (30%, 3/10) reported that patients stay less than 1 h, 40%, (4/10) reported 1 to 2 h, and 20% (2/10) reported more than 2 h.

## Discussion

In this study, we noted several key findings: (1) As expected, UC facility hours are typically longer than standard outpatient office visit hours, with the vast majority open in the evenings and a significant minority open on weekends, and lengths of stay are typically quite short (7/9 reporting 2 h or less); (2) The majority of the UC facilities surveyed offer intravenous treatments, and those that offered intravenous treatments offered at least one of the level B recommended drugs for migraine management in the acute care setting: IV metoclopramide or IV prochlorperazine; (3) Half of those surveyed had headache treatment protocols but the majority of the UC facilities do not have follow-up protocols in place; (4) Most UC physicians have EM, IM or Family Medicine Training.

Long hours of operating at UCs provide a person with migraine with the option to seek care outside of the emergency department after-hours. In addition to the cost benefits outlined above, urgent care settings are quieter and less crowded, factors that are important to a patient in the midst of an intractable migraine. Furthermore, emergency department wait times tend to be longer and parking more distant, and such delays are not only inconvenient, but also delay relief from migraine. As migraine is a chronic disorder with episodic attacks, patients are likely to seek care for an intractable episode if the visit is focused, convenient, and provides quick effective relief.

The majority of the UC facilities surveyed offer intravenous treatments, with at least one of the level B recommended drugs: IV metoclopramide or IV prochlorperazine. (Currently, there are no Level A recommended medications for the acute management of migraine in the emergency setting.) Per the American Headache Society guidelines, Level B medications should be offered to patients for acute migraine treatment based on available evidence [15].

Half of those surveyed had headache treatment protocols and the majority of the UC facilities did not have follow-up protocols in place. Many did not have a protocol in place for diagnostic workup or referral to higher level care like the ED, as only 2 programs reported referral to ED. They also did not have protocols for how to best treat the patients in the UC or upon discharge. Previous studies have shown that patients with migraine visiting UC are not receiving treatment according to the highest level of evidence [11]. In addition, the route of administration is critical, with oral medications often failing to address the headache at home, parenteral treatments may be warranted. However, only 3 out of 10 medications reportedly offered at UCs in the survey were available by parenteral route. The following adjustments may help to improve diagnosis, promote individualized care, and increase use of medications of the highest level of evidence − 1) use of a validated screening tests such as ID Migraine to assist with diagnosis of migraine, 2) widespread use of the Migraine Action Plan [13, 16] would allow the patient’s outpatient headache provider to identify a personalized approach to acute headache management in the UC setting, 3) employing an algorithm or protocol for headache management within and upon discharge from UC. An example of such algorithms includes those used in the ED which have helped to reduce the use of opiates in that setting [17]. Migraine infusion protocols, if put in place, may allow for better utilization of existing resources at urgent care clinics. This would include expansion of other therapies felt to benefit migraine, including, but not limited to: fluids, ketorolac, magnesium sulfate, valproic acid, and corticosteroids [18]. The UCs had opioids, and use of protocols may limit use of opioids for headache management. UC facilities serve as an emergency bridge to provide a temporary care between the patient and primary neurologist or headache expert. Patients should return to their outpatient provider(s) for continuity of care as soon as possible for optimization of both preventive and abortive treatment. A post discharge satisfaction survey from UC may also be helpful to further improve care.

### Future work in urgent care

As stated in the prior paper on UC visits for headache in NYC, regulation or standardization of UC facilities varies across states, so we sought to better understand how urgent care centers outside of NYC operate and might manage headache/migraine. As this is a newly expanding area with potential for headache management, there are several areas for potential study (See Table 6).

### Strengths

This is the first study examining how urgent care centers in various parts of the country may be used for headache/migraine and provides a glimpse into how they may or may not have the ability to offer intravenous medications, have certain medications in stock, or have protocols already in place for headache.

### Limitations

This study had several limitations. Our QI project utilized convenience sampling. This may have assisted with obtaining data from multiple regions of the US, nevertheless, we cannot generalize our findings to all urgent care centers. Future research could include randomized trials with increased and randomized sampling for greater generalizability.

Moreover, this is a QI study and is not generalizable to the whole population. The data was collected through clinician reported surveys, thus incorporating recall and estimation biases that may have impacted the analysis. Future studies may consider prospective data collection to limit the impact of these biases. During the assessment of treatment protocols we did not explore the use of distinct protocols for types of acute headaches (for example: migraine and non-migraine common primary headache disorders) We did not examine provider level data or patient level data to examine whether there were transfers to EDs, the reasons for the transfers, etc. Patient level data, (e.g. clinical outcomes, demographics, etc.) of those treated for headache disorders in UCs may enrich our understanding of UC role in the treatment of headache disorders and help to examine impact of healthcare utilizations and costs. We also did not obtain volume data for the UC sites. Patient volume data may inform the systematic role and impact of UC on headache care in the US. Our work is solely a glimpse into considerations for how the headache community might consider working with UC facilities, considerations for discussion and considerations for future research.

## Conclusion

Limited access to quality care is a significant contributor to gaps in US healthcare. The limited number of clinicians with expertise in headache management, together with the limited options for acute headache management within the confines of a typical outpatient clinic (with or without infusion capabilities), forces patients to seek care in the ED. UC centers are traditionally less busy than EDs and, with their ability to provide care during extended hours, can prove to be a valuable accommodation for patients needing management of acute headaches. Patients with predictable clinical presentations and responses to previously tried abortive regimens (for example, acute migraine) may benefit the most. Our study aimed at exploring the current infrastructure and practice parameters at UC centers as it pertains to managing acute headaches in adults and the results are very informative. A larger-scale study may provide further insight in this regard, and the preparedness for UC facilities to develop headache-specific protocols and provide quality care for headache patients. While there is a need for the development of clinical guidelines and evidence-based approaches specific to UC centers to improve outcomes, we implore on the proposition to build partnerships with UC centers with the goal of providing value-driven care that is timely and effective.

## Supplementary Information

**Additional file 1.**

## Data Availability

Data can be shared in accordance with NYU’s data sharing policies. Please contact the study PI, Mia T. Minen, MD, MPH at minenmd@gmail.com if you would like to request data.

## Abbreviations

AHS: American Headache Society
ED: Emergency Department
IRB: Institutional Review Board
NP: Nurse Practitioner
PCP: Primary Care Practitioner
QI: Quality Improvement
UC: Urgent Care
