# Supplementary material for: The role of urgent care centers in headache management: a quality improvement project
Source: BMC Health Serv Res. 2022 Feb 8;22:162. doi: 10.1186/s12913-021-07457-2 (PMC8822636; doi:10.1186/s12913-021-07457-2)
Supplement: Supplementary file 1 — Additional file 1. [file 12913_2021_7457_MOESM1_ESM.docx]

**APPENDIX**

**Urgent Care Centers and Headache Management**

**Date:** ((D-M-Y))

**Name:** (First and Last)

Affiliation (hospital, healthcare network, etc.):

How many urgent care sites are affiliated with your hospital or in your surrounding area*?*

Urgent Care Center and location:

(If multiple, please list)

Please specify location area:

Urban

Suburban

Rural

Other

How many patient visits are there each year?

*A*re they free standing or part of your institution if you belong to a medical institution?

Please specify:

*We are interested in finding out more about staffing at urgent care centers. Please consider the following with regard to urgent care staff:*

**What are the # of MD*s/*DOS:**

*W*hat residency training was completed?

Emergency medicine

Family medicine

Internal medicine

Other

Other: (Please Specify)

*W*hat are the prerequisites or qualifications for them to work there?

Do they administer IM medications?

yes

no

Do they administer IV medications?

yes

no

Are there any Physician Assistants (PAs) at this site?

yes

no

**What are the # of PAs*?***

What are the prerequisites or qualifications for them to work there?

((e.g. need to have had 2 years of work experience post residency training))

Do they administer IM medications?

yes

no

Do they administer IV medications?

yes

no

Are there NPs at this site?

yes

no

**What are the # of NPs?**

What are the prerequisites or qualifications for them to work there*?*

((e.g. need to have had 2 years of work experience post residency training))

Do they administer IM medications?

yes

no

Do they administer IV medications?

yes

no

**Are there nurses*?***

yes

no

If so, how many?

If possible, please describe nurse staffing e.g. one nurse per two medical providers (MD*/DO/*NP*/*PA):

What are their responsibilities? Please explain:

**What are the *#* of Medical assistants (MAs)?**

*W*e are also interested in finding out more about general operations at urgent care centers. Please consider the following with regard to hours, operations, and protocols:

What are the hours of the urgent care centers by day?

Please provide a value range (ex: 8am-4pm)

Monday:

Tuesday:

*W*ednesday:

Thursday:

Friday:

Saturday:

Sunday:

Is it regular practice for providers (any) at your urgent care to do pain checks?

yes

no

Are there any visual analog scales or other pain assessments used in evaluating and managing patients presenting with headache to the urgent care(s)?

yes

no

If yes, which pain assessments are used?

Are there any diagnostic test(s) your urgent care performs onsite for patients with headache disorders*?*

yes

no

Please specify which tests:

How is migraine (or headache disorder) diagnosed at the urgent care locations?

Do they use "ID migraine", ICHD3 or other clinical diagnostic tools?

yes

no

Which diagnostic tools do they use?

Is there a headache or migraine protocol?

yes

no

If possible, please upload a copy of the protocol as this would be extremely helpful.

**If unable to get the migraine*/*headache protocol:**

What are the medications*/*treatments in your migraine*/*headache protocol or therapy plan?

Are there any policies in place to ensure follow-up with a patient's PCP, neurologist, or headache specialist?

yes

no

If so, please describe:

Which migraine medication(s*)*/anti-emetic(s) do they keep in their pharmacy? Select all that apply:

Metoclopramide IV, Metoclopramide PO, Prochlorperazine IV, Depakote IV , Dihydroergotamine (DHE) IVF, Diphenhydramine IV, Magnesium IV, Ketorlac I*V,* Dexamethasone IV, Ondansetron (PO) O, Sumatriptan INJ, Oral triptans, Morphine IV O, Morphine PO, Dilaudid IV, Percocet*/*tylenol #3, Ibuprofen PO, Naprosyn PO, Acetaminophen IV, Acetaminophen PO, Ketamine IV O, Ketamine NS

Other

If other, please specify:

Any reported information on average length of stay for "headache" and*/*or "migraine" patients? Please specify (in hours):

Are there any publicly available statistics on approximately what percentage of patients that present with headache have a disposition to the emergency department?

Select which types of providers you refer to:

Primary Care Physician (PCP)

Neurologist O Headache Specialist

Pain Specialist Other Healthcare provider

If other healthcare provider, please specify:

Are there home urgent care centers in your area?

yes

no

Please write a detailed summary of the data you can find out about them and whether they treat headache*/*migraine or not.

Please add any additional comments you think would be useful for this study:
